# TGF-β1-induced bone marrow mesenchymal stem cells (BMSCs) migration via histone demethylase KDM6B mediated inhibition of methylation marker H3K27me3

**DOI:** 10.1038/s41420-022-01132-z

**Published:** 2022-07-28

**Authors:** Qiting He, Jie Shi, Wei Liu, Wei Zhao, Zihao Wang, Kaiwen Liu, Dawang Zhao, Shaoyi Wang, Yongyuan Guo, Lei Cheng, Yuan Gao

**Affiliations:** 1 Department of Orthopedic Surgery, Qilu Hospital of Shandong University, Cheeloo College of Medicine, Shandong University, 250012 Jinan, Shandong China; 2 NHC Key Laboratory of Otorhinolaryngology, Qilu Hospital of Shandong University, Cheeloo College of Medicine, Shandong University, 250012 Jinan, Shandong China

**Keywords:** Mesenchymal stem cells, Mesenchymal migration

## Abstract

Mesenchymal stem cells (MSCs) are widely used in clinical research and therapy. Since the number of MSCs migration is extremely crucial at the lesion site, exploring the mechanisms to enhance the migration of MSCs is necessary. Therefore, this study focused on the epigenetic mechanisms in MSCs migration. TGF-β1 stimulated bone marrow mesenchymal stem cells (BMSCs) to promote cell migration at lesion sites in vitro and in vivo. The mRNA and protein levels of several migration-related genes (N cadherin, CXCR4, FN1) were enhanced. The trimethylation marker H3K27me3 recruitment on the promoter of these genes were studied to dissect the epigenetic mechanisms. TGF-β1 elevated the levels of KDM6B leading to removal of repression marker H3K27me3 in the promoter region of N cadherins and FN1. Congruently, knockdown of demethylase KDM6B substantially affected the TGF-β1 induced BMSCs migration. This promoted the down-regulation of various migration-related genes. Collectively, epigenetic regulation played an important role in BMSCs migration, and H3K27me3 was at least partially involved in the migration of BMSCs induced by TGF-β1.

## Introduction

Bone marrow mesenchymal stem cells (BMSCs) have the ability to differentiate into osteoblasts, chondrocytes and adipocytes under specific conditions that provide an opportunity to treat various bone defects, cartilage defects and osteoporosis [1–3]. The migration of an adequate amount of BMSCs to the lesion site is the essential requirement for the treatment with good efficacy. However, the specific mechanism of BMSCs migration and homing is complicated and elusive [4, 5]. Therefore, it brings the demand to conduct the deeper study of the mechanism involved in BMSCs migration to establish a stronger foundation for the clinical applications.

Transforming growth factor-β1 (TGF-β1) is a multifunctional protein that can regulate the growth, differentiation, migration, apoptosis and immune regulation of a variety of cells [6, 7]. TGF-β1 promotes migration of several kinds of cell types such as tumor cells, nerve cells, endothelial cells, MSCs and so on [8–10]. TGF-β1 is one of the most abundant cytokines in the bone matrix which allow BMSCs recruitment at the area of damage for bone reconstruction [11, 12]. It has been reported that TGF-β1 promotes BMSCs to migrate to the site of myocardial ischemia in rats through SDF1/CXCR4 pathway [13]. There are several evidence suggesting TGF-β1 releases from bone resorption sites to initiate BMSCs migration to bone resorption sites through SMAD signaling pathway to promote bone formation and maintain bone stability [14]. However, more detailed studies are required to have better understanding of the complex process BMSCs migration and the TGF-β1 role.

Histone methylation is an important chromatin modification regulating epigenetics and involved in several functions as heterochromatin formation, gene expression, X chromosome inactivation and transcription regulation [15]. Histone demethylases (LSD1 and JmjC family demethylases) are a group of enzymes that catalyze the removal of the methyl group from histones [16]. The demethylation of histones is involved in the regulation of gene transcription and expression, which involve many biological processes [17]. Despite the fact that histone modifications are some good marks for transcriptional states, it is still strongly debated for many marks whether they are causing activation/repression or there to ensure memory of transcriptional states, recent studies indicated some histone marks associated with active genes do not directly cause transcriptional activation [18]. Some previous studies have reported that trimethylation of lysine residue 27 on histone 3 (H3k27me3) inhibits the gene expression whereas removing this methylation mark to promote gene expression via chromatin interactions [19, 20].

Histone demethylases (KDM6A and KDM6B) is known to mediate the removal of the methylation mark of H3k27me3, which involved in many physiological and disease processes [17, 21]. Some studies have shown that histone demethylases are involved in cell differentiation, KDM4B and KDM6B play major roles in osteogenic differention of MSCs [22]. There are evidence suggesting the role of histone demethylase KDM6B in cell migration by removing methyl groups from histones and thereby, regulating the expression of cell migration-related genes. Overexpression of histone demethylase KDM6B enhances tumor cell migration and stem cell-like traits [23]. Jumonji domain-containing protein 3, also called as histone demethylase KDM6B, is involved in vascular remodeling where vascular smooth muscle cells proliferate and migrate to form intimal hyperplasia post blood vessels injury [24]. There is very limited knowledge regarding the epigenetics modifications of TGF-β1 in homing of BMSCs. This certainly points to conduct deeper studies on the effect of epigenetics on BMSCs migration for clinical applications. Since KDM6B plays a certain role in the migration of other cells, we studied the role of KDM6B in TGF-β1 induced BMSC migration to better explain the mechanism of epigenetics in BMSC migration.

Our findings explored the effect of TGF-β1 on BMSCs migration in vivo and in vitro. Additionally, we also decipher the role of histone demethylase KDM6B and the H3K27me3 level in TGF-β1-mediated BMSCs migration. This study brings light on the epigenetic mechanisms involved in BMSCs homing mediated through chromatin modification of TGF-β1 from epigenetics.

## Results

### TGF-β1 induces BMSCs migration in vitro

We started by exploring the effect of TGF-β1 on BMSCs migration. We observed an enhanced migration of BMSCs at 24 h upon 5 ng/ml TGF-β1 stimulation by performing scratch test (Supplementary Fig. 1A, B). Consequently, transwell analysis confirmed a significant increase in BMSCs migration upon TGF-β1 induction at 24 h (Supplementary Fig. 1C, D).

### TGF-β1 promotes the expression of KDM6B in BMSCs

We investigated the impact of TGF-β1 on histone demethylase by qPCR analysis. We found that histone demethylase, KDM6B noticeably upregulated compared to KDM4A, KDM4B and KDM6A (Supplementary Fig. 2A) and subsequently observed the upregulation in the mRNA and protein levels of migration-related genes (N cadherin; CXCR4: CXC motif chemokine receptor type 4; FN1: fibronectin 1) upon TGF**-**β1 stimulation (Supplementary Fig. 2B–D and Supplementary Fig. 3A). The increased expression of CXCR4 by TGF**-**β1 was further validated by immunofluorescence (IF) (Supplementary Fig. 2E, F). Moreover, TGF-β1 promoted F-actin reorganization of BMSCs and increased number of actin cortical protrusions (Supplementary Fig. 2G, H).

### Silencing of KDM6B inhibits the migration of BMSCs in vitro

**Fig. 1:**
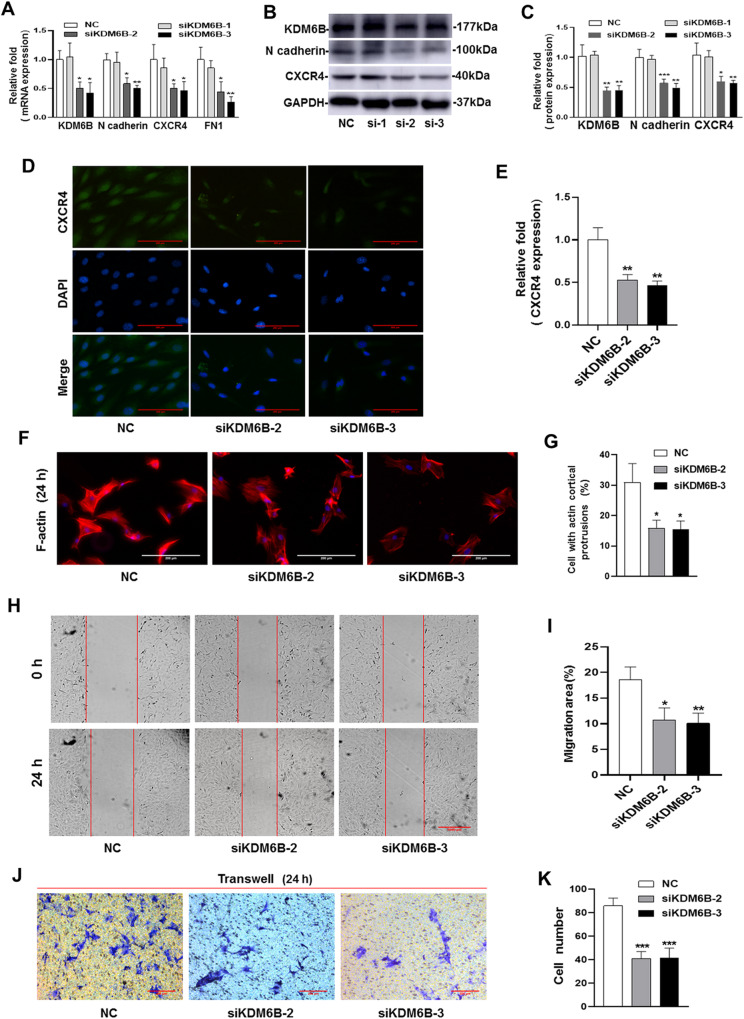
The siRNA-KDM6B inhibited the migration of BMSCs in vitro. **A** The knockdown efficiency of KDM6B was verified by qRT-PCR. **B**, **C** The siRNA-KDM6B inhibited the protein expression of migration-related genes (N cadherin and CXCR4). **D**, **E** The IF showed the expression of CXCR4 was inhibited by siRNA-KDM6B. **F**, **G** The siRNA-KDM6B inhibited the actin cortical protrusions formation of BMSCs. **H**, **I** The scratch test showed that siRNA-KDM6B decreased the migration area of BMSCs. **J**, **K** Transwell test verified that siRNA-KDM6B inhibited the migrated MSCs number. (si-1: siKDM6B-1, si-2: siKDM6B-2, si-3: siKDM6B-3. ^*^P < 0.05, ^**^P < 0.01, ^***^P < 0.001 in comparison with the control group; ^##^*P* < 0.01, ^###^*P* < 0.001 in comparison with TGF**-**β1 group. All experiments were repeated three times independently. All error bars ± standard deviation.).

To study the role of KDM6B in BMSCs migration, we used three independent small interfering RNAs (siRNA) to silence KDM6B confirmed by qRT-PCR and western blotting with 60% knockdown (siKDM6B-2, siKDM6B-3), moreover, the expression of various migration-related genes namely, N cadherin, CXCR4 and FN1 were downregulated upon silencing of KDM6B, however, the role of siKDM6B-1 is not significant (Fig. 1A–C, Supplementary Fig. 3B). Given the potential off-target effects, we performed the following experiments using two independent siRNAs (siKDM6B-2, siKDM6B-3). The IF results of CXCR4 showed the expression of CXCR4 was inhibited by siRNA-KDM6B (Fig. 1D, E). Moreover, the knockdown of KDM6B inhibits cortical protrusions formation in BMSCs (Fig. 1F, G). Most importantly, scratch test showed that the migration area of BMSCs is significantly reduced upon KDM6B silencing (Fig. 1H, I) and congruently, transwell assay showed decreased cell migration (Fig. 1J, K).

### Silencing of KDM6B inhibits the migration of BMSCs promoted by TGF-β1

**Fig. 2:**
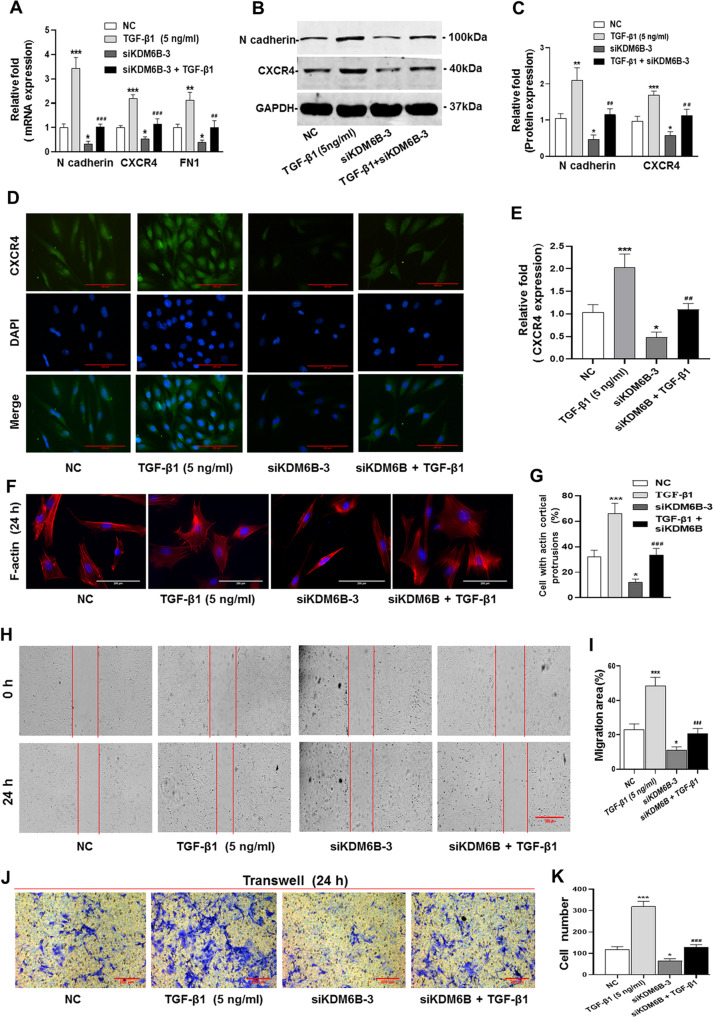
Silencing of KDM6B inhibits the migration of BMSCs promoted by TGF-β1. **A**–**C** The siRNA-KDM6B inhibited the increased mRNA and protein expression level of migration-related genes promoted by TGF**-**β1. **D**, **E** The IF showed the CXCR4 expression in presence of TGF-β1 upon siRNA-KDM6B. **F**, **G** The siRNA-KDM6B significantly inhibited the actin cortical protrusions formation promoted by TGF-β1. **H**, **I** The scratch test showed that siRNA-KDM6B decreased the migration area of BMSCs, and also inhibited the migration area promoted by TGF-β1. **J**, **K** Transwell test verified that siRNA-KDM6B inhibited the migrated MSCs number promoted by TGF-β1. (^*^P < 0.05, ^**^P < 0.01, ^***^P < 0.001 in comparison with the control group; ^##^*P* < 0.01, ^###^*P* < 0.001 in comparison with TGF**-**β1 group. All experiments were repeated three times independently. All error bars ± standard deviation.).

To study the role of KDM6B in BMSCs migration induced by TGF**-**β1, we used siKDM6B-3 to test the inhibition role of silencing of KDM6B in the migration of BMSCs promoted by TGF-β1. The results of qRT-PCR and western blotting showed the expression of various migration-related genes (N cadherin, CXCR4 and FN1) promoted by TGF**-**β1 stimulation were downregulated upon silencing of KDM6B (Fig. 2A–C and Supplementary Fig. 3C). The IF results of CXCR4 are similar with qPCR and western blotting (Fig. 2D, E). Moreover, the knockdown of KDM6B inhibits cortical protrusions formation in BMSCs promoted by TGF**-**β1 (Fig. 2F, G). Most importantly, wound-healing and migration assays strongly suggested KDM6B involvement in the migration of BMSCs by TGF**-**β1. Scratch test showed that the migration area of BMSCs induced by TGF**-**β1 is significantly reduced upon KDM6B silencing (Fig. 2H, I) and similarly, transwell assay showed decreased cell migration number (Fig. 2J, K). The migration ability promoted by TGF**-**β1 were significantly inhibited by siRNA-KDM6B. These results suggested that KDM6B is plausibly involved in BMSCs migration by TGF**-**β1.

### Histocompatibility of BMSCs and PLGA scaffold, release study of TGF-β1 from the scaffold

**Fig. 3:**
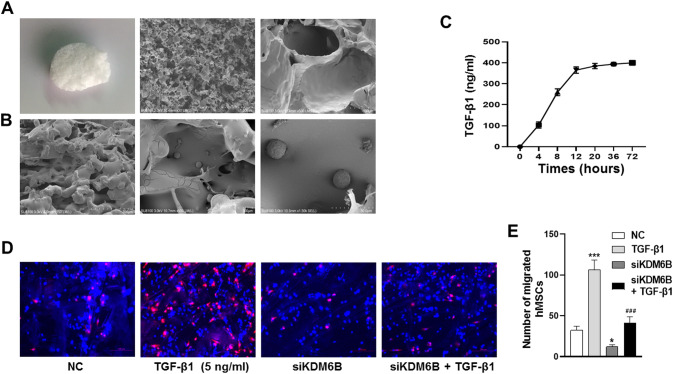
The siRNA-KDM6B inhibited BMSCs homing promoted by TGF-β1 in vivo. **A** The appearance shape and internal microstructure SEM of PLGA scaffold. **B** BMSCs are adhered on the scaffolds under SEM. **C** The efficiency of TGF**-**β1 released from PLGA scaffold. **D** The CM-Dil labeled BMSCs migrated to PLGA scaffold in vivo. **E** The relative quantitative of migrated BMSCs. (^*^P < 0.05, ^***^P < 0.001 in comparison with the control group; ^###^*P* < 0.001 in comparison with TGF**-**β1 group. All experiments were repeated three times independently. All error bars ± standard deviation.).

To further study the role of KDM6B in the migration of BMSCs promoted by TGF-β1 in vivo, we used poly (lactic-*co*-glycolic acid) (PLGA) scaffold loaded TGF-β1 to perform the experiments in vivo. Firstly, we tested the histocompatibility of BMSCs and PLGA scaffold, and release study of TGF-β1 from the PLGA scaffold in vitro. The appearance and internal microstructure of PLGA scaffold were studied by SEM (Fig. 3A). The strong adhesion between PLGA scaffolds and BMSCs was observed suggesting a desirable biocompatibility (Fig. 3B). TGF**-**β1 was efficiently released, verified by concentration quantification, near to 400 ng/ml at 12 h (Fig. 3C). These results indicated PLGA scaffolds are very biocompatible with BMSCs, and TGF**-**β1 is efficiently released from PLGA scaffolds.

### The knockdown of KDM6B inhibits BMSCs homing promoted by TGF-β1 in vivo

PLGA scaffolds were used to study the homing of BMSCs in vivo. The results showed that TGF-β1-loaded PLGA scaffolds has more accumulation of CM-Dil labeled BMSCs and other vivo cells on day 7, suggesting that TGF-β1 has the ability to induce BMSCs migration and homing in vivo. The migrated ability of BMSCs and aggregation in the PLGA scaffolds were greatly reduced upon silencing of KDM6B despite TGF-β1 induction (Fig. 3D, E).

### TGF-β1 promotes the gene expression by removing H3K27me3 in the promoter region through KDM6B

**Fig. 4:**
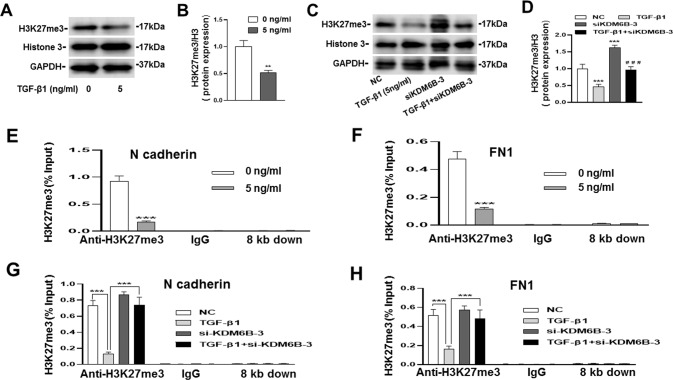
TGF-β1 regulated the gene expression by removing H3K27me3 in the promoter region through KDM6B. **A**–**D** The results of western blotting showed that the protein expression of H3K27me3 was downregulated by TGF-β1, but this inhibitory effect can be partially reversed by silencing of KDM6B. **E**, **F** The levels of H3K27me3 in the promoter regions of N cadherin and FN1 were removed by TGF-β1, IgG and 8 kb downstream of the transcription start site were used as negative control. **G**, **H** The siRNA-KDM6B could reverse the decreased levels of H3K27me3 at the promoter regions of N cadherin, and FN1 caused by TGF-β1. (^**^P < 0.01, ^***^P < 0.001 in comparison with the control group, ^###^*P* < 0.001 in comparison with TGF**-**β1 group. All experiments were repeated three times independently. All error bars ± standard deviation.).

**Fig. 5:**
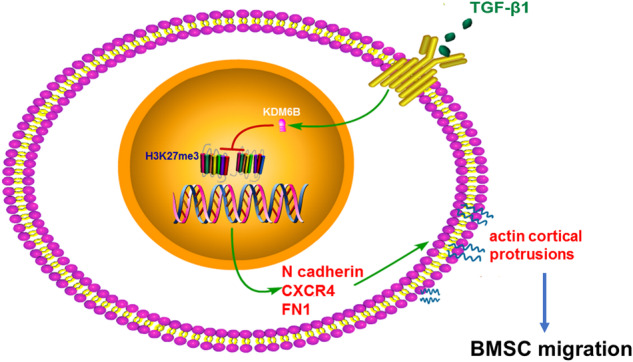
Mechanism diagram of TGF-β1-induced BMSCs migration. A summary diagram for TGF-β1 induced BMSCs migration via histone demethylase KDM6B mediated inhibition of methylation marker H3K27me3.

In order to verify that TGF-β1 regulates the expression of migration-related genes through epigenetics, we performed CHIP-qPCR to analyze H3K27me3 enrichment at the promoter regions of N cadherin and FN1. The western blotting results showed that the protein expression of H3K27me3 was downregulated by TGF-β1, but this inhibitory effect can be partially reversed by silencing of KDM6B (Fig. 4A–D and Supplementary Fig. 3D). It was evidently observed that TGF-β1 removed H3K27me3 in the promoter region of N cadherin and FN1 through KDM6B (Fig. 4E, F). Contrastingly, the repression marker H3K27me3 increased upon KDM6B silencing which downregulated the expression of N cadherin and FN1 (Fig. 4G, H). However, H3K27me3 in the promoter of CXCR4 was not detected, this suggested that KDM6B does not directly regulate the expression of CXCR4. Figure 5 is a summary diagram for TGF-β1 induced BMSCs migration via histone demethylase KDM6B mediated inhibition of methylation marker H3K27me3.

## Discussion

BMSCs are multifactorial cell types with capability to promote bone repair, cartilage repair and tissue regeneration [25–27]. The recruitment of BMSCs to the lesion area is the foci of treatment. The therapeutic effect is measured through number of BMSCs migration to the site of lesion [28]. TGF-β1, as a member of the super family of transforming factors, can induce the migration of a variety of cells. Many studies have been reported directing that TGF-β1 can induce the migration of MSC in vitro and in vivo. The study has shown that TGF-β1 in the prostate cancer tissue induced MSC migration into cancerous tissue and activated cancer-associated fibroblasts [29]. There are pieces of pieces of evidence demonstrated the necessity of noncanonical signals and N-cadherin for TGF-β1-induced BMSCs migration [30]. However, the role of epigenetic mechanisms in BMSCs migration is not fully understood. The trimethylation marks on histones regulates the expression of genes where H3K27me3 is popularly known to be involved in inactivation of transcription [31, 32]. On the other hand, the histone demethylation enzyme KDM6B removes the trimethylation marks on histones and promotes gene expression [33]. Therefore, we intended to explore the contribution of epigenetics in the migration mechanisms of BMSCs and provide a reference for clinical therapy.

Our findings suggest that TGF-β1 promotes the upregulation of histone methylase namely KDM4B and KDM6B which are directly proportional to the expression of migration-related genes (N cadherin, FN1 and CXCR4) and actin cortical protrusions of BMSCs. Most importantly, the enrichment of H3K27me3 in the DNA promoter regions of N cadherin and FN1 was significantly reduced. In addition, scratching and transwell tests further confirmed that TGF-β1 promoted the migration of BMSCs, and in vivo experiments confirmed that TGF-β1 could recruit BMSCs. Upon KDM6B silencing, dramatic down-regulation in the expression of N cadherin, FN1 and CXCR4, inhibition in the TGF-β1 induced migration and increased enrichment of H3K27me3 in the promoter region of N cadherin and FN1, were observed. In vivo experiments further strengthened our in vitro findings when the migration was dramatically reduced upon KDM6B knockdown.

Histone demethylase KDM6B can remove the H3K27me3 mediated trimethylation marks and regulate the expression of related genes [34–36]. Similar to our results, TGF-β1 can promote the expression of KDM6B, and then regulate gene expression [37, 38]. In auto-immune disease like rheumatoid arthritis, KDM6B induces proliferation and migration of fibroblast-like synoviocytes, which are involved in joint destruction and pathologic processes [39]. It has been observed that H3K27me3 loss elevates the migration of tumor cells, the secretion of various stem cell niche factors and the metastasis of breast cancer [20, 40]. Adenosine monophosphate-activated protein kinase (AMPK) enhances cdx2 expression by inhibiting H3K27me3 accumulation at the promoter and thereby, induces epithelial cell migration and intestinal barrier function [41].

TGF-β1 can regulate the expression of a variety of migration-related genes, including N cadherin, FN1, MICAL1, MICAL2, MICALL2 and so on [42]. N cadherin is involved in the cell growth, migration, and differentiation processes by mediating cell-to-cell interactions, which is also important for the migration of BMSCs [43–45]. Studies have shown that BMSCs with high expression of CXCR4 promoted enhanced migration ability to bone marrow [46]. FN1 is involved in cell adhesion and migration and provides a key substrate for cell migration via hydrolyzing protein fragments to promote cell chemotactic migration [47, 48].

Considering all the evidence produced, our study positively suggests that histone demethylase KDM6B removes methylation marks at N cadherin and FN1 promoters and promotes TGF-β1 induced BMSCs migration. This study enriched the role of epigenetics in cell migration.

## Materials and methods

### The extraction and culture of BMSCs

The experiments were undertaken with the understanding and written consent of each patient, the study methodologies conformed to the standards set by the Declaration of Helsinki, and were approved by Shandong University ethics committee. The femoral heads and necks were obtained from patients who underwent hip replacement surgery (average age: 50–60 years). The bone marrow was collected with sterile vascular clamp and placed into sterile PBS (Beyotime, Shanghai, China). It was minced into small pieces using with repetitive washes and finally, collected the suspension into 50 ml centrifuge tube (NEST, Jiangsu, China) which was centrifuged for 5 minutes at 1000 r/min. The PBS was discarded and bone marrow cells pellet was resuspended into the complete medium (α-MEM (Gibco, USA) with 10% fetal bovine serum (Gibco) and 1% penicillin-streptomycin antibiotic (Gibco)). The cells were seeded into a 25 cm^2^ culture flask and cultured in a CO_2_ incubator at 37 °C. The medium was replaced to remove unattached cells at day 3 followed by replenishing every 3 days. Once the confluency 90%, cells were passaged at 1:2 ratios. BMSCs from passage 3 to 5 were used for subsequent experiments.

### Wound-healing and migration assays

For the wound-healing assay, hBMSCs were seeded in a 6-well plate (2 × 10^5^ cells/well). Once the transfected cells reached its maximum confluency, three parallel scratches were made at the bottom of the dish using 200 µl tips. The suspended cells were washed off using PBS. The medium containing 0.5% FBS with or without TGF-β1 (5 ng/ml, peprotech, USA) was used to stimulate cells for 24 h. The same scratch area was photographed by Lionheart FX (Bio Tek, USA) at 0 h and 24 h. ImageJ software was employed to analyze the efficiency of cell migration.

For the migration assay, the transfected cells (5 × 10^4^ cells) were seeded in the upper chamber in 200 µl serum-free medium and 600 µl medium containing 10% FBS was added to the lower chamber with or without TGF-β1 (5 ng/ml). After 24 h, the cells were fixed with 4% polyformaldehyde (Solarbio, Beijing, China) for 15 min at room temperature and washed with PBS thrice. 0.1% crystal violet (Solarbio) was used to stain cells for 30 mins at room temperature and later was washed with PBS 3 times. Residual crystal violet was removed by gentle wiping using cotton bar. Inverted microscopy (Leica DMI4000 B) was used to capture migrated cells and the number of migrated cells were measured by ImageJ.

### RNA extraction and quantitative real-time polymerase chain reaction (qRT-PCR)

For the qRT-PCR, BMSCs were seeded into 6-well plate (2 × 10^5^ cells/well). According to the experimental protocol, total RNA was extracted using Trizol reagent (TIANGEN, Beijing, China) and the Nanodrop Lite (ThermoFisher Scientific, USA) was used to measure RNA concentration. The RNA was reverse transcribed into cDNA following the instructions in the ReverTra Ace Qpcr RT Kit (Toyobo Life Science, Shanghai, China). The expression levels of target genes were detected by qRT-PCR on ABI 7900HT (ThermoFisher Scientific) using SYBR Green Realtime PCR Master Mix (Toyobo Life Science). The detailed primer sequences of genes are shown in Table 1, and glyceraldehyde-3-phosphate dehydrogenase (GAPDH) was used as an internal reference to normalize the expression of each gene.

**Table 1:** Sequences of the primers.

Gene	Forward (5′–3′)	Reverse (5′–3′)
*N cadherin*	TCCTGCTTATCCTTGTGCTGA	AAAAGTTGTTTGGCCTGGCG
*CXCR4*	TGGTCTATGTTGGCGTCTGG	GTCATTGGGGTAGAAGCGGA
*FN1*	AGCCGAGGTTTTAACTGCGA	CCCACTCGGTAAGTGTTCCC
*GAPDH*	TCATGGGTGTGAACCATGAGAA	GGCATGGACTGTGGTCATGAG

*CXCR4* CXC motif chemokine receptor type 4, *FN1* fibronectin 1, *GAPDH* glyceraldehyde-3-phosphate dehydrogenase

### Western blotting

BMSCs were seeded into 6-well plate (2 × 10^5^ cells/well). Cells were lysed using RIPA lysate (Beyotime) and protease inhibitor cocktail (MedChemExpress, Shanghai, China) on the ice. The protein concentration was measured using the BCA kit (BOSTER, Wuhan, China). Each sample (40 µg) was analyzed by 10% SDS-PAGE gel (Beyotime) and transferred to a PVDF membrane (Merck Millipore, Shanghai, China). The membrane was sealed by 5% skim milk at room temperature for 1 h followed by TBST washes thrice for 5 min each. The PVDF membrane was incubated overnight at 4 °C with the following primary antibodies: KDM6B (1:1000, DF13101, Affinity Biosciences, Jiangsu, China), N cadherin (1:1000, AF5239, Affinity Biosciences), CXCR4 (1:1000, AF5279, Affinity Biosciences), GAPDH (1:5000, AF7021, Affinity Biosciences). Next day, the primary antibodies were recycled and membrane was washed with TBST for 3 times for 10 minutes each. The anti-rabbit IgG (H + L) (DyLight™ 680 Conjugate) (1:10000, CST, Shanghai, China) was used as secondary antibody at room temperature for 30 min. After three washes with TBST, the protein expression level was detected by Odyssey two-color infrared fluorescence imaging system (LI-COR, USA) and measured by ImageJ.

### Transfection of siRNA-KDM6B

Cells were seeded into 6-well plate (2 × 10^5^ cells/well). The synthesized siRNA-KDM6B (siRNA-KDM6B-1, 2, 3) and negative control (NC) were obtained from the GenePharma company (Shanghai, China). When the cells reached the confluency of 60–80%, transfection was performed using NC and siRNA-KDM6B-1, 2, 3 using micropoly-transfecter^TM^ cell reagent (Micropoly Biotech, Jiangsu, China). The fresh medium was replaced after 8 h of transfection. Total RNA and protein were extracted after 24 h and 48 h to perform qRT-PCR and western blotting, respectively. The detailed sequences of NC and siRNA-KDM6B are shown in Table 2.

**Table 2:** Primer sequences of siRNA-KDM6B.

Gene	Sense primer (5′–3′)	Anti-sense primer (5′–3′)
*NC*	UUCUCCGAACGUGUCACGUTT	ACGUGACACGUUCGGAGAATT
*si-KDM6B-1*	GGAUGGAGAGAUCUUAGAATT	UUCUAAGAUCUCUCCAUCCTT
*si-KDM6B-2*	GGGUUCUCAUGGUGCUCAAAC	UUGAGCACCAUGAGAACCCGG
*si-KDM6B-3*	GAGACCUCGUGUGGAUUAATT	UUAAUCCACACGAGGUCUCTT

### F-actin staining of BMSCs with phalloidin

BMSCs (5 × 10^4^ cells/well) were seeded in a 24-well plate (NEST). Upon reaching 60% confluency, the cells were transfected with NC or siKDM6B for 6 h. The cells were then stimulated with or without TGF-β1 (5 ng/ml) in a fresh medium for 24 h. Followed by 4% paraformaldehyde (Solarbio) fixing, the cells were incubated with 100 nM phalloidin in dark to stain F-actin for 30 min at room temperature. The nucleus was stained with DAPI (4′,6-diamidino-2-phenylindole, Beyotime) for 5 min. Images were captured using an inverted microscope (Leica DMI4000 B) where BMSCs with cortical protrusions were identified as positive cells. 30 cells were randomly observed.

### Cell immunofluorescence (IF)

The cells (5 × 10^4^ cells/well) were seeded over the sterile coverslips (Diameter: 14 mm, NEST) placed at the bottom of the 24-well plate. On reaching 60% confluency, the cells were transfected with NC or siKDM6B for 6 h. The medium was changed to fresh medium with or without TGF-β1 (5 ng/ml). Fixation, permeabilization and blocking were performed post 24 h according to the protocol of cell immunofluorescence. BMSCs were incubated with anti-CXCR4 (1:100, Affinity Biosciences) immunofluorescent antibody at 4 °C overnight followed by incubation with immunofluorescent secondary antibody (1:100, Abbkine, Wuhan, China) at room temperature for 1 h. After cell nucleus were stained with DAPI for 5 min, cell fluorescence was captured by an inverted microscope (Leica DMI4000 B) and the relative expression of CXCR4 was quantified by ImageJ.

### Chromatin immunoprecipitation (CHIP)

For the CHIP assay, BMSCs (6 × 10^5^) were seeded into 10 cm tissue culture dish (NEST). Transfection was performed according to the above protocol. After 12 h stimulation with or without TGF-β1, the chromatin of the cells was extracted according to the CHIP kit (EZ-Magna ChIP TM A/G, Merck Millipore, USA). One part of chromatin was used as input, one part was mixed with anti-IgG antibody and the remaining part was mixed with 7 µl anti-H3K27me3 antibody (ab6002, Abcam, USA). The qPCR was performed using the eluted DNA to analyze the enrichment of H3k27me3 in the DNA promoter regions of N cadherin, CXCR4 and FN1. Detailed primer sequences are shown in Table 3.

**Table 3:** Primer sequences of promoter region.

Gene	Forward (5′–3′)	Reverse (5′–3′)
*N cadherin*	ACAAAGAGCAGCAGTCCCG	GAACAGTCTCCAACTCGCCG
*8* *kb downstream*	ATTTATGTTAAGCGGGAGCGG	GGGTGGATACACTTGGCTTGA
*CXCR4*	CCAGCAAATAAGCCCGGAGA	AAAATCGTGGAAGACGCCGA
*8* *kb downstream*	TTGCTGGGACTCTTGCATCT	GGCAGAGCCAGTCCACTATT
*FN1*	ATTGCGTCACCTCTCTTCGG	GAGTCCCGAGTCAGTACCCT
*8* *kb downstream*	CAGTCACCTTTAGATCTCTCCC	AATGATGTACCAGGCTTGGGTT

*CXCR4* CXC motif chemokine receptor type 4; *FN1* fibronectin 1

### Histocompatibility of BMSCs and materials, release study of TGF-β1

The PLGA scaffold was obtained from Jinan Daigang Biomaterial company (Shandong, China). After disinfection, the cells (2 × 10^5^/well) were seeded on the PLGA scaffold (1 × 1 × 0.3 cm^3^) in a 24-wells plate. After 24 h, PLGA scaffolds were fixed using the electron microscope fixing solution (Solarbio). The morphology and histocompatibility of PLGA scaffold were observed and photographed under scanning electron microscopy (SEM, Germany).

To study the secretion of TGF-β1 from the PLGA scaffold, TGF-β1 (5 µg) was loaded into PLGA scaffold, and soaked with 1 ml PBS in a 24-well plate. The concentration of TGF-β1 was detected by BCA kit (BOSTER) at 4 h, 8 h, 12 h, 20 h, 36 h, and 72 h.

### BMSCs homing study in vivo

8 weeks old nude mice were purchased from the SPF (Beijing) Biotechnology Company (Beijing, China) and were randomly divided into four groups as the NC group, si-KDM6B group, TGF-β1 group and TGF-β1 + si-KDM6B group with each group containing four mice, all procedures were approved by the Shandong University ethics committee on the Use and Care of Animals and complied with all relevant animal welfare laws, guidelines and policies. After successful anesthesia, an incision (about 1.5 cm) was made on the back to expose the muscle tissue. PLGA scaffold with or without TGF-β1 was placed in the muscle space and the incision was sewed layer by layer. Transfected BMSCs (6 × 10^6^ cells) labeled by CellTracker^TM^ CM-Dil Dye (Invitrogen, USA) according to the protocol, were injected through the tail vein. After 7 days, the nude mice were sacrificed and PLGA scaffolds were removed, fixed, frozen section, DAPI staining, and observed under an inverted fluorescence microscope (Leica DMI4000 B).

### Statistical analysis

All the quantitative data were expressed as mean ± standard deviation, and all experiments were independently repeated at least 3 times, Student’s *t* test was selected for two groups and one-way ANOVA was selected for more than 2 group comparisons. GraphPad software (version 8.0.2) was used to analyze statistical differences of data, *p* < 0.05 represented the difference was statistically significant.

## Supplementary information

Supplementary information

Supplementary Figure 1

Supplementary Figure 2

Original Data File

Supplementary Figure 3

## Data Availability

The data that support the findings of this study are available from the corresponding author upon reasonable request.
