# Supplementary material for: TGF-β1-induced bone marrow mesenchymal stem cells (BMSCs) migration via histone demethylase KDM6B mediated inhibition of methylation marker H3K27me3
Source: Cell Death Discov. 2022 Jul 28;8:339. doi: 10.1038/s41420-022-01132-z (PMC9334584; doi:10.1038/s41420-022-01132-z)
Supplement: Supplementary file 1 — Supplementary information [file 41420_2022_1132_MOESM1_ESM.docx]

**Supplementary Fig. 1 TGF-β1 promoted BMSCs migration in vitro. A)** The scratch test of BMSCs at 0 h and 24 h. **B)** The relative quantitative of migration area. **C)** The transwell assay of BMSCs at 0 h and 24 h. **D)** the relative quantitative of migrated BMSCs. (***P* < 0.01, ****P* < 0.001 in comparison with the control group. All experiments were repeated three times independently. All error bars ± standard deviation.)

**Supplementary Fig. 2 TGF-β1 promoted the expression of KDM6B in BMSCs. A)** TGF-β1 promoted the mRNA expression of histone demethylase KDM6B. **B-D)** The relative mRNA and protein expression level of migration related genes induced by TGF-β1. **E-F)** The IF and relative expression quantitative of CXCR4. **G-H)** TGF-β1 promoted the actin cortical protrusions formation of BMSCs. (**P* < 0.05, ***P* < 0.01, ****P* < 0.001 in comparison with the control group. All experiments were repeated three times independently. All error bars ± standard deviation.)
