# Supplementary figures and images for: TGF-β1-induced bone marrow mesenchymal stem cells (BMSCs) migration via histone demethylase KDM6B mediated inhibition of methylation marker H3K27me3
Source: Cell Death Discov. 2022 Jul 28;8:339. doi: 10.1038/s41420-022-01132-z (PMC9334584; doi:10.1038/s41420-022-01132-z)

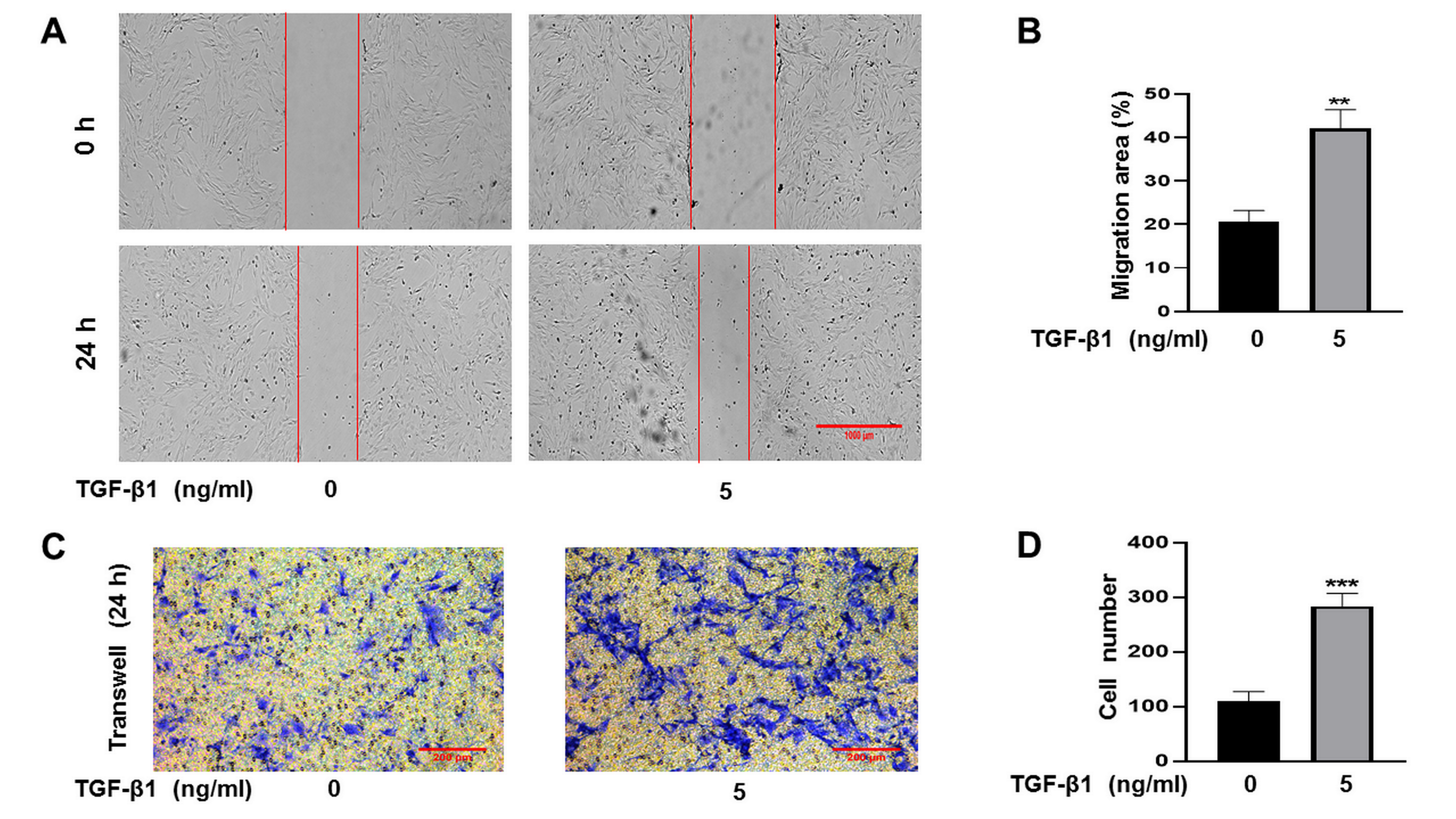

Supplement: Supplementary file 2 — Supplementary Figure 1 [file 41420_2022_1132_MOESM2_ESM.tif]

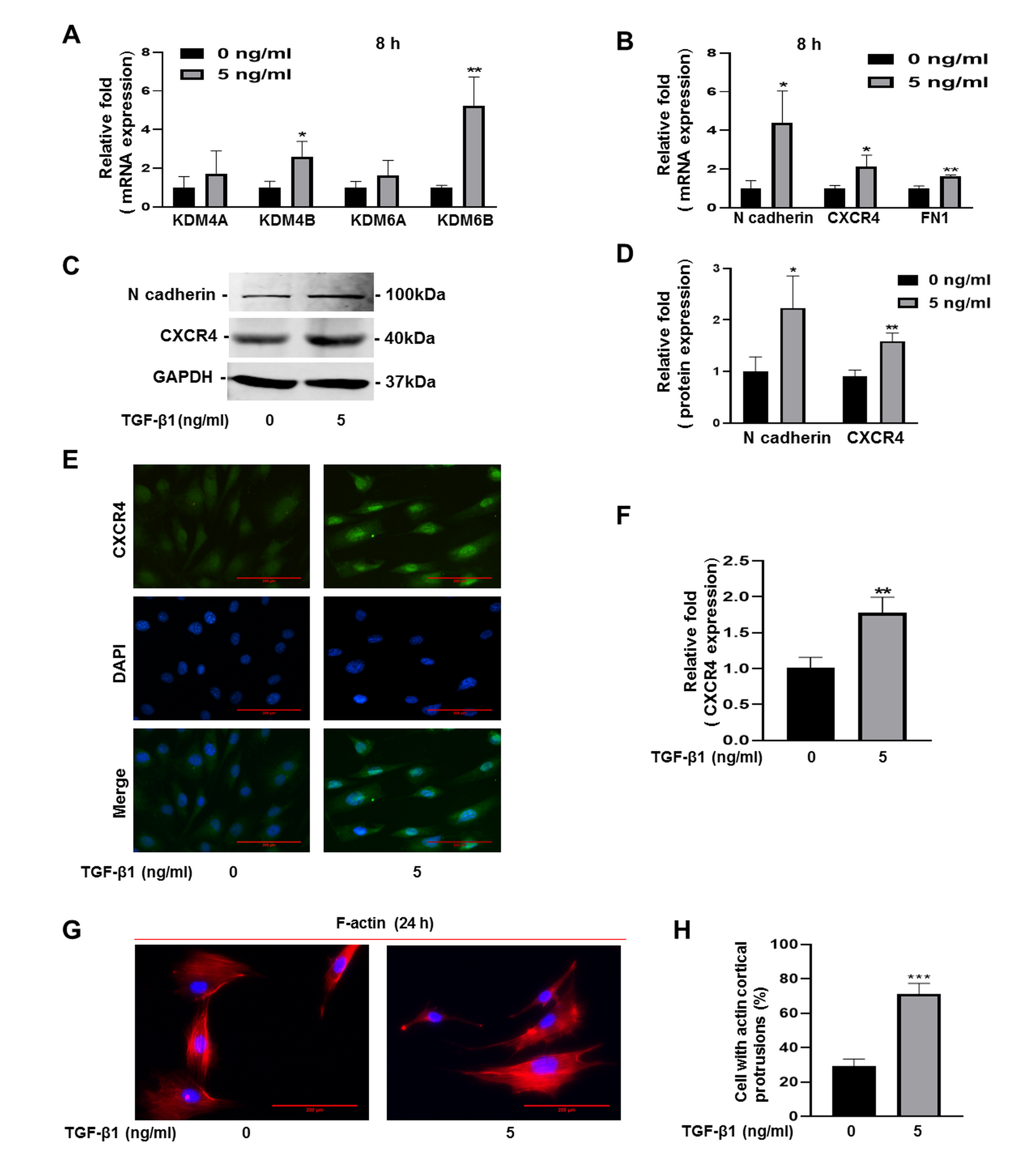

Supplement: Supplementary file 3 — Supplementary Figure 2 [file 41420_2022_1132_MOESM3_ESM.tif]

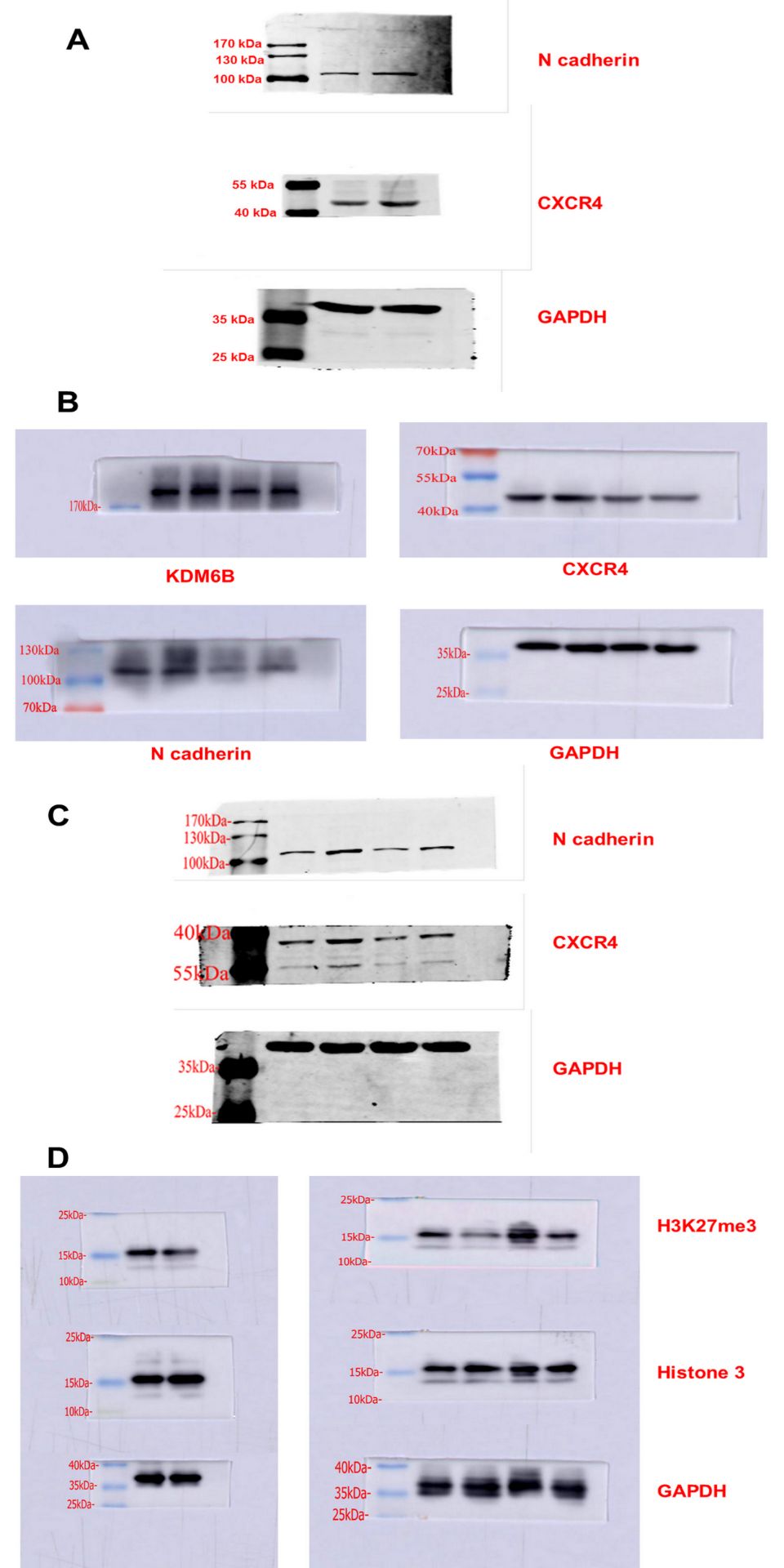

Supplement: Supplementary file 5 — Supplementary Figure 3 [file 41420_2022_1132_MOESM5_ESM.jpg]
