# Supplementary material for: TGF-β1-induced bone marrow mesenchymal stem cells (BMSCs) migration via histone demethylase KDM6B mediated inhibition of methylation marker H3K27me3
Source: Cell Death Discov. 2022 Jul 28;8:339. doi: 10.1038/s41420-022-01132-z (PMC9334584; doi:10.1038/s41420-022-01132-z)
Supplement: Supplementary file 4 — Original Data File [file 41420_2022_1132_MOESM4_ESM.docx]

**A**


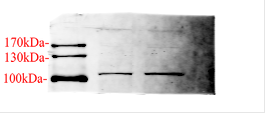


**N cadherin**


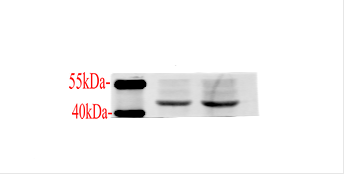


**CXCR4**


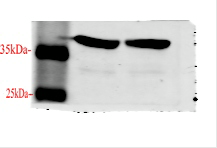


**GAPDH**

**B**


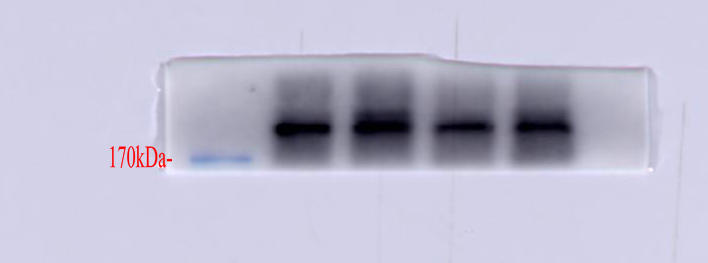

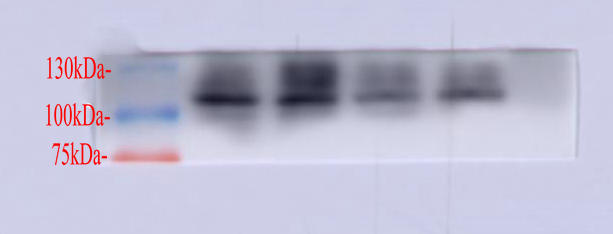

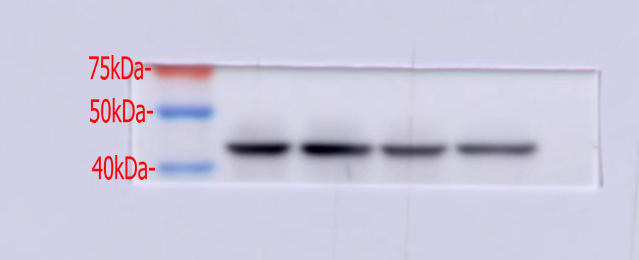

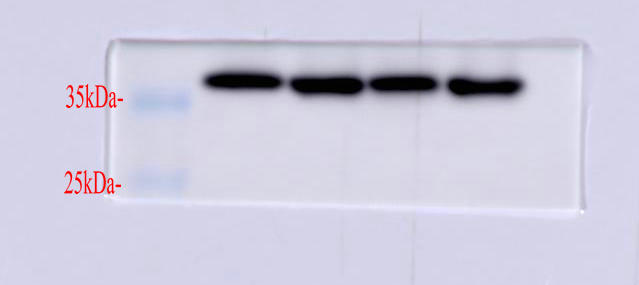


**CXCR4**

**GAPDH**

**N cadherin**

**KDM6B**

**C**


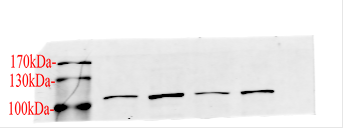


**N cadherin**


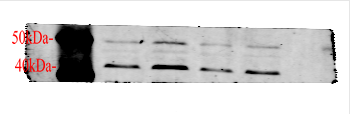


**CXCR4**


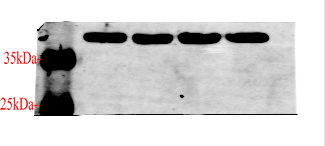


**GAPDH**

**D**


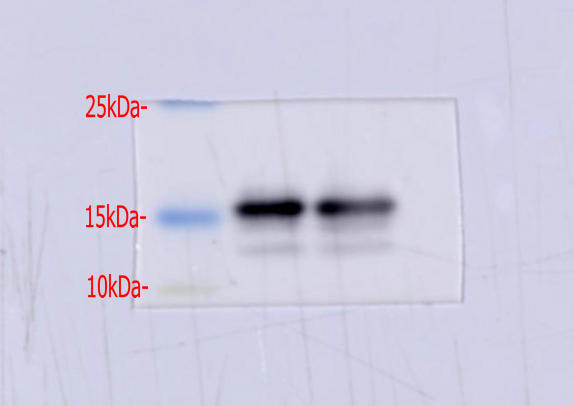

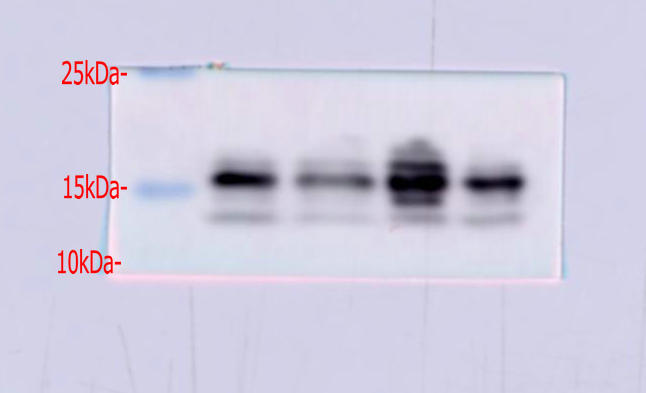


**H3K27me3**


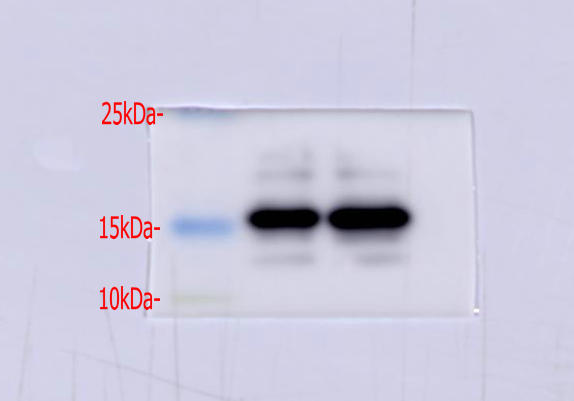

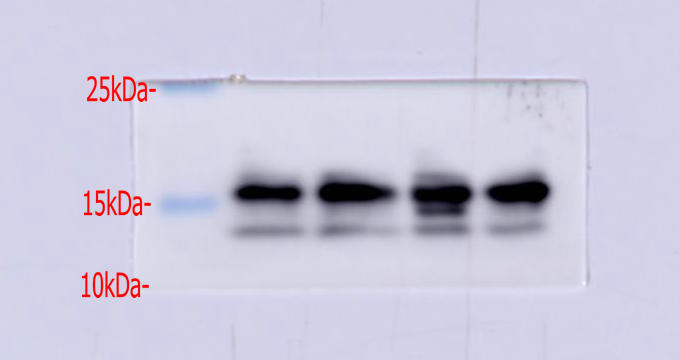


**H3**


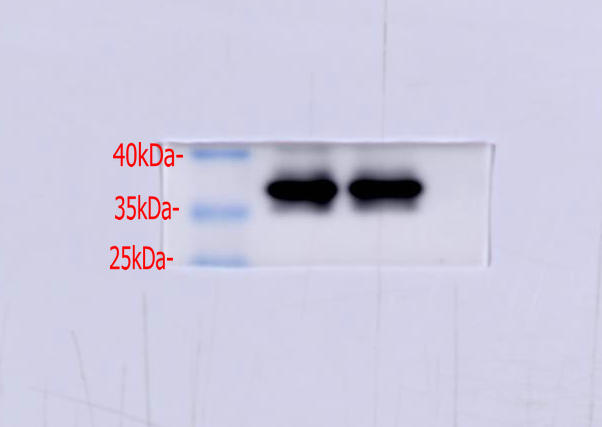

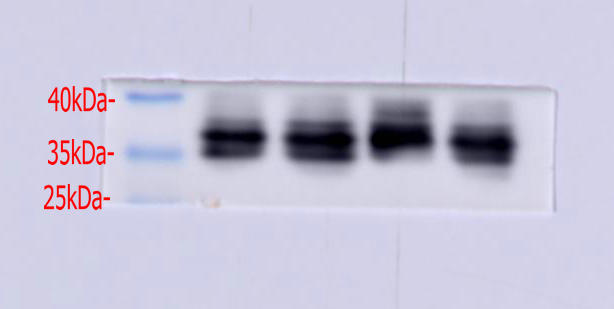


**GAPDH**

**N cadherin**
